# Fast Selective Detection of Pyocyanin Using Cyclic Voltammetry

**DOI:** 10.3390/s16030408

**Published:** 2016-03-19

**Authors:** Fatima AlZahra’a Alatraktchi, Sandra Breum Andersen, Helle Krogh Johansen, Søren Molin, Winnie E. Svendsen

**Affiliations:** 1 Department of Micro- and Nanotechnology, Technical University of Denmark, Ørsteds Plads, 2800 Kgs. Lyngby, Denmark; Winnie.Svendsen@nanotech.dtu.dk; 2 Novo Nordisk Foundation Center for Biosustainability, Technical University of Denmark, Kogle Allé 6, 2970 Hørsholm, Denmark; sandrabreumandersen@gmail.com (S.B.A.); hkj@biosustain.dtu.dk (H.K.J.); sm@bio.dtu.dk (S.M.); 3 Department of Systems Biology, Technical University of Denmark, Kemitorvet, 2800 Kgs. Lyngby, Denmark; 4 Department of Clinical Microbiology, Afsnit 9301, Rigshospitalet, Juliane Maries Vej 22, 2100 København, Denmark

**Keywords:** electrochemical detection, pyocyanin, quorum sensing, diagnosis, cyclic voltammetry

## Abstract

Pyocyanin is a virulence factor uniquely produced by the pathogen *Pseudomonas aeruginosa*. The fast and selective detection of pyocyanin in clinical samples can reveal important information about the presence of this microorganism in patients. Electrochemical sensing of the redox-active pyocyanin is a route to directly quantify pyocyanin in real time and *in situ* in hospitals and clinics. The selective quantification of pyocyanin is, however, limited by other redox-active compounds existing in human fluids and by other metabolites produced by pathogenic bacteria. Here we present a direct selective method to detect pyocyanin in a complex electroactive environment using commercially available electrodes. It is shown that cyclic voltammetry measurements between −1.0 V to 1.0 V reveal a potential detection window of pyocyanin of 0.58–0.82 V that is unaffected by other redox-active interferents. The linear quantification of pyocyanin has an *R*^2^ value of 0.991 across the clinically relevant concentration range of 2–100 µM. The proposed method was tested on human saliva showing a standard deviation of 2.5% ± 1% (*n* = 5) from the known added pyocyanin concentration to the samples. This inexpensive procedure is suggested for clinical use in monitoring the presence and state of *P. aeruginosa* infection in patients.

## 1. Introduction

*Pseudomonas aeruginosa* is reported to be among the most problematic bacteria in infections such as bloodstream, surgical wound, burn wound, and cystic fibrosis lung infections [1,2,3]. This opportunistic pathogen is seldom responsible for infections in healthy individuals, but is successful in immune-compromised patients, where infection causes significant morbidity and mortality [4,5]. While factors causing the infections to occur are well known, little is documented about the signals that allow benign bacteria to become pathogenic [6]. Thus, information regarding the pathogenic behavior of *P. aeruginosa* can be gained by monitoring the initial signals produced by this bacterium.

Special for *P. aeruginosa* is the production of the redox-active molecule pyocyanin. Pyocyanin production is controlled by quorum sensing [7]. *P. aeruginosa* uses quorum sensing to make collective decisions about virulence expression. Pyocyanin is assumed to be released prior to virulent activity and may itself be a quorum sensing signal [8,9]. Thus, the ability to assess the pyocyanin level produced in patients can reveal valuable information about the state of progression of the infection before a clinical infection is apparent. The redox-active nature of pyocyanin which is uniquely produced by a problematic bacterium such as *P. aeruginosa* makes it an excellent biomarker to determine whether a patient is in danger.

In the clinic, body fluids such as urine, blood and sputum are used to determine if a patient is infected with bacteria. This is done via a microbiological culture of a sample from the body fluid to verify if an infection is present. Sputum cultures are specifically used to help identify the types of infections in the lungs and airways of cystic fibrosis patients. Sputum is normally not produced by healthy individuals, but can be produced in small quantities if irritation of the airways occurs, such as in the case of smokers and asthma patients. The presence of bacteria in sputum implies that it is possible to detect pyocyanin directly in sputum samples from patients. However, this also means that pyocyanin exists in a background of complex body fluids and this needs to be taken into account when performing measurements.

Currently, the detection of pyocyanin is accomplished by high-performance liquid chromatography (HPLC) or spectrophotometry. However, these are mostly time-consuming and costly approaches due to the pre-purification of samples. They also require isolation and culturing of bacterial samples. Electrochemical sensing is an increasingly popular method for the measurement of biochemical compounds due to the ability of specific and sensitive detection of desired molecules [10]. One of the advantages of electrochemical sensors is that they can be incorporated into point-of-care (POC) devices, providing fast and real-time diagnosis of the infection state in patients without any pre-treatment [11].

It is possible to detect pyocyanin by electrochemical sensors due to its redox-active nature. There have only been a few reports on detecting pyocyanin using electrochemistry. Sharp *et al.* presented a carbon fiber sensor for the electrochemical sensing of pyocyanin capable of detecting pyocyanin concentrations between 1 µM and 100 µM [12]. Webster and Goluch were able to detect pyocyanin with palladium hydride reference electrodes integrated in a microfluidic up-concentration device [13]. More recently, Sismaet *et al.* detected pyocyanin produced by *P. aeruginosa* by biochemically up-regulating the pyocyanin production [14]. These methods are starting to make their way into the goal of clinical detection of pyocyanin in patients, some of them also by using commercially available electrodes [15]. It is common for the studies that detect pyocyanin electrochemically to find that pyocyanin is exclusively detected at negative potentials around −250 mV to −300 mV, claiming that no other chemicals can interfere with this signal [16,17]. Conversely, interferents from dead cells in human fluids can be released and detected at negative potentials, creating misleading results that could falsely be identified as pyocyanin. In reality, pyocyanin is produced in an environment of redox-active precursors and metabolites that, from an electrochemical perspective, are close to pyocyanin [18,19,20]. Hence, the way to clinical diagnosis of infections using pyocyanin as an infection biomarker starts with the ability to selectively detect pyocyanin among interfering compounds.

The results from this study provide a specific method to selectively detect pyocyanin in a complex mixture of interacting compounds using relatively high positive potentials. In addition, this method has been applied on human saliva samples spiked with pyocyanin. The closest body fluid to sputum is saliva, which has been used in the model of this study. The Department of Clinical Microbiology at Rigshospitalet in Copenhagen has shown that only 50% of all sputum samples are representative of the actual lower airway condition of the patients. The sputum samples were mixed with the saliva produced by the patient, which makes saliva important to characterize before any attempt of direct pyocyanin detection in sputum samples from patients [21]. With background in the results of this study, we suggest using this method for early infection diagnostics in sputum from patients with lung infections.

## 2. Experimental Section

### 2.1. Reagents

Stock solutions of 100 µM pyocyanin (P0046-25MG, SIGMA, Copenhagen, Denmark), pyoverdine (P8374-1MG, SIGMA, Copenhagen, Denmark), NADP (N5755 SIGMA, Copenhagen, Denmark), NADPH (N5130 SIGMA, Copenhagen, Denmark), NAD (N1636, SIGMA-ALDRICH, Copenhagen, Denmark), NADH (N4505, SIGMA, Copenhagen, Denmark), phenazine-C_12_H_8_N_2_ (P13207 ALDRICH, Copenhagen, Denmark) and Lysogeny Broth (LB) medium were prepared in MilliQ water, respectively.

### 2.2. Preparation of Samples

Dilutions between 1 µM and 100 µM were prepared for all the compounds. A mixture of the mentioned compounds was prepared containing 100 µM of each compound including pyocyanin. This mixture will be referred to as mix-1. A corresponding mixture without addition of pyocyanin was also prepared, and referred to as mix-2. A dilution series in the range between 0 µM and 100 µM pyocyanin was prepared in MilliQ water and in background concentrations of 5, 25, 50 and 100 µM mix-2, respectively.

### 2.3. Cyclic Voltammetry Measurements—Experimental Protocol

Disposable screen-printed electrodes with a three-electrode configuration were used for the electrochemical experiments (C223AT, Dropsens, Spain). The electrodes consisted of a 1.6 mm gold working electrode, a gold counter electrode and a silver reference electrode. The electrodes were connected to a potentiostat (Metrohm Autolab, The Netherlands) from which cyclic voltammetry was used to characterize the electrochemical profile of the different compounds and to quantify the pyocyanin content. The software Autolab NOVA 1.10 (Metrohm Autolab, The Netherlands) was used for data handling, peak finding and analysis.

All the compounds were individually characterized by five cyclic voltammetry sweeps between −1.0 V to 1.0 V and sweep rates of 0.05 V/s using concentrations of 100 µM of each compound. 

Cyclic voltammograms between −1.0 V to 1.0 V and a scan rate of 0.05 V/s were used to characterize the profiles of mix-1 and mix-2. The calibration curve was obtained by detecting the current difference for the peaks of cyclic voltammograms performed on the dilution series of pyocyanin and mix-1, respectively; using a potential window of −0.4 V to 1.0 V and a scan rate of 0.05 V/s. All the measurements were conducted *versus* the Ag reference electrode.

### 2.4. Cyclic Voltammetry of Human Saliva and Artificial Sputum

Human saliva (approximately 2 mL) was collected from healthy volunteers fasting 12 h prior to the experiment. The saliva was placed on the electrodes and all air bubbles were gently removed. A cyclic voltammetry scan from −1.0 V to 1.3 V was performed using a scan rate of 0.10 V/s. Saliva was mixed with 100 µM mix-2 and 160 µM pyocyanin and placed on new electrodes before measuring the current in a potential window of −1.0 V to 1.0 V using a scan rate of 0.10 V/s. This was repeated five times.

Artificial sputum (ASM) was prepared according to Kirchner *et al.* [22]. A cyclic voltammetry scan from −1.0 V to 1.0 V was performed using a scan rate of 0.10 V/s. All the measurements were conducted *versus* the Ag reference electrode.

## 3. Results and Discussion

### 3.1. The Selectivity Window of Pyocyanin

**Figure 1:**
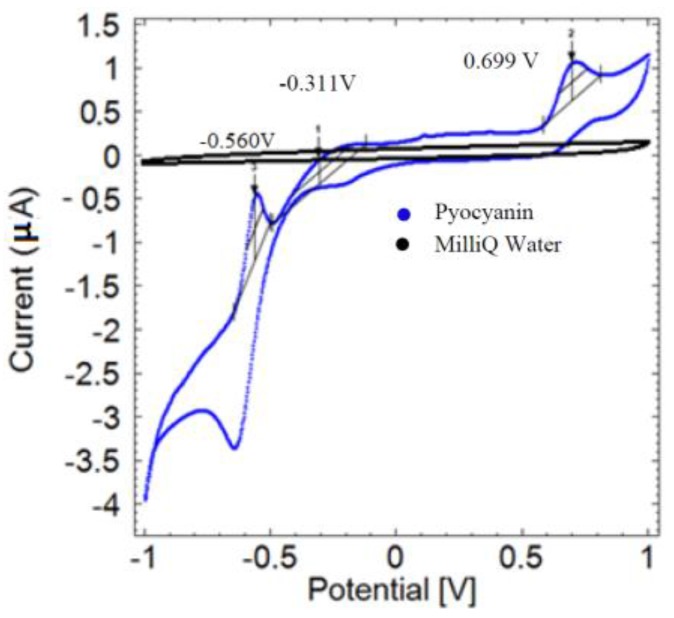
Cyclic voltammogram of 500 µM pyocyanin (blue) compared to the MilliQ water control (black) using a scan rate of 0.05 V/s. No signals are generated from cyclic voltammetry of MilliQ water, while pyocyanin reveals a characteristic profile with oxidation peaks at −0.560 V, −0.311 V and 0.699 V (arrows) measured *versus* the Ag reference electrode.

The preliminary assessment of the electrochemical profile of pyocyanin using cyclic voltammetry shows several oxidation peaks at −0.560 V, −0.311 V and 0.699 V (Figure 1, blue curve). The MilliQ water control gave no peaks, as expected (black curve). It is likely that the peak at −0.311 V is equivalent to the pyocyanin peaks located around −0.25 V to 0.30 V reported by earlier studies [6,12,13].

**Figure 2:**
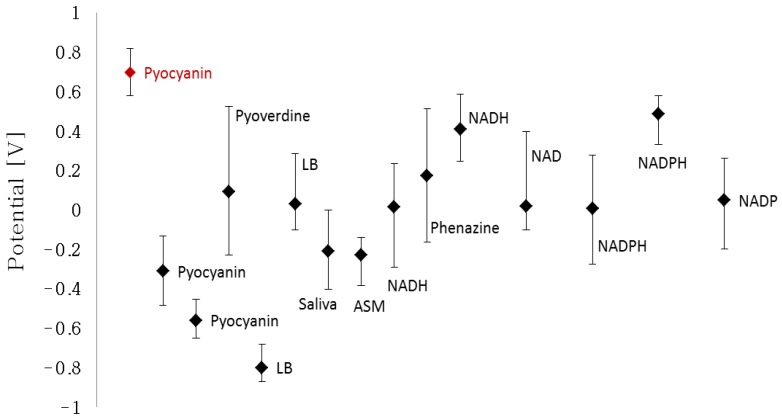
Potential peak values of the different compounds and related detection width (bars) extracted from cyclic voltammograms of the individual compounds. The detection window of pyocyanin with peak at 0.699 V and corresponding start and end potentials lies outside the interaction width of the other redox-active compounds.

The characteristic peak potentials of NAD, NADH, NADP, NADP, pyoverdine, phenazine, LB, human saliva and ASM were extracted from their respective cyclic voltammograms (Figure 2). The individual cyclic voltammograms of the different compounds can be viewed in the Supplementary data. The detection window of each compound was determined by the characteristic potential peak that increases proportionally with the increasing concentration. The bars represent the lower and higher potential intervals which the max peak lies within. Several compounds are represented twice as two or more peaks corresponded to the increasing concentration. Figure 2 shows that pyocyanin has two detection windows that are located outside the characteristic detection windows of the interfering compounds, namely at −0.560 V and at 0.699 V. In contrast, the window of detection with a max peak at −0.311 V overlaps with human saliva and ASM. This implies that it will not be possible to distinguish if a peak in this potential window belongs to pyocyanin or the tested body fluid in a cyclic voltammogram. The implication of this interference is the main reason why the relatively positive peak potential is more beneficial to use in the quantification experiments compared to the negative peak potential. The unique and independent detection window of pyocyanin between 0.58 V and 0.82 V allows selectivity in detection among the other redox-active chemicals. 

As the max potential peak of 0.699 V was chosen for further quantification, a suitable scan range from −0.4 V to 1.0 V was established for the following quantitative investigations. This relatively high potential has never been applied in pyocyanin quantification in previous research. Therefore, the electrochemical behavior of pyocyanin at these relatively high potentials in a mixture of redox-active compounds is not yet available for clinical use. The increase of electrode potential is a self-enhancing process. When the electrode potential is increased, the ohmic potential drops and the signal-to-noise ratio is enhanced. Thus, the use of relatively high characteristic potentials might enhance the limit of detection of pyocyanin, when considering use of, e.g., square wave voltammetry or amperometry.

### 3.2. Identifying Electrochemical “Fingerprint” of Pyocyanin among Interacting Compounds

**Figure 3:**
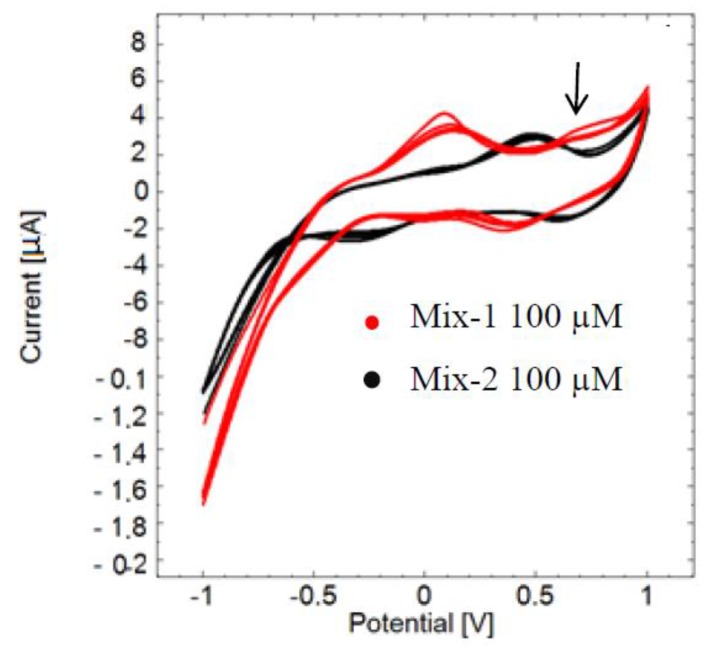
Cyclic voltammograms of mixture of compounds containing pyocyanin (mix-1, red curve) and mixture without pyocyanin (mix-2, black curve) measured *versus* the reference electrode. The red curve has a peak at 0.68 V confirming the presence of pyocyanin (arrow), while it is absent in the black curve of mix-2.

A clear electrochemical fingerprint of pyocyanin was observed when detected among other redox-active compounds that were equal in concentration to pyocyanin. The cyclic voltammogram of mix-1 clearly reveals the presence of pyocyanin in the sample as a peak at 0.68 V (Figure 3, red curve). On the other hand, no pyocyanin peak is apparent in the cyclic voltammogram of mix-2 (black curve). Mix-2 gives a superposition of an oxidation peak at 0.48 V and a width that is slightly overlapping with the pyocyanin detection window. In mix-1 it seems likely that the superposition of the peaks of the interfering compounds has shifted to peak around 0.10 V, which is close to the picture given by Figure 2. Another remarkable observation is that the potential window with a peak at −0.560 V detected in the cyclic voltammogram of pure pyocyanin (Figure 1 and Figure 2) did not appear in Figure 3. This may be due to a non-reversible oxidation of pyocyanin.

### 3.3. Detection Limit of Pyocyanin in a Complex Mixture of Interfering Compounds

**Figure 4:**
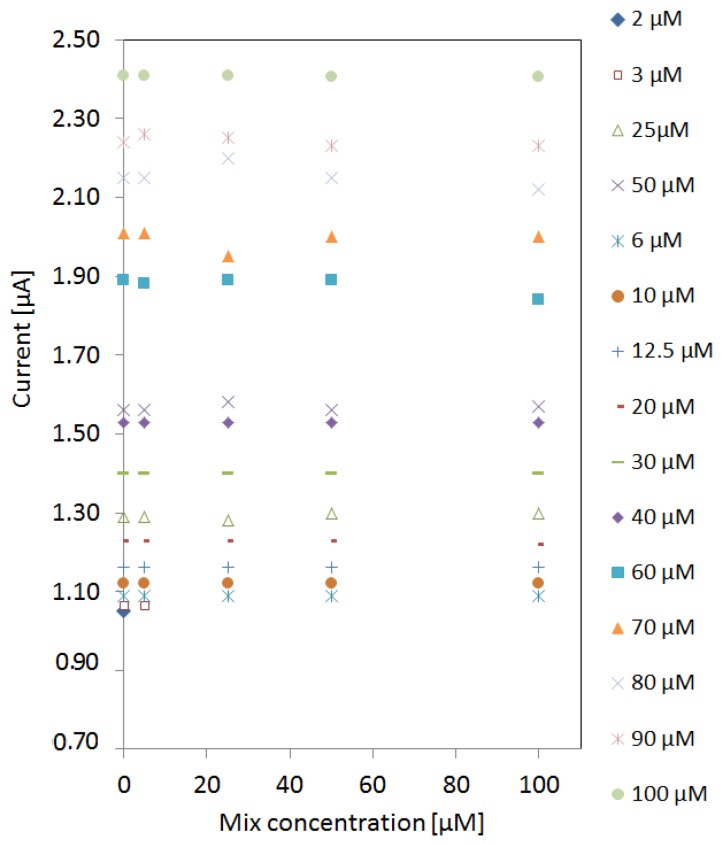
Detection of pyocyanin in different mix backgrounds between 5 µM and 100 µM in addition to pyocyanin detection in MilliQ water (zero mix background). The current peaks of the varying pyocyanin concentrations were unaffected by the mix concentration. Detection of 2 µM pyocyanin was only possible when no interfering signals were present. When the mix concentration was above 25 µM the detection limit of pyocyanin remained 6 µM.

After characterizing the signature of pyocyanin among the interferents, the limit of detection (LOD) was investigated. A new sensor was used for each measurement to avoid cross-contamination or fouling of electrodes. The peak currents in the detection window of pyocyanin were extracted from cyclic voltammograms of pyocyanin in different mix concentrations as presented in Figure 4. The peak currents were stable despite the increase of mix concentration. However, the LOD of pyocyanin was affected by the presence of the increasing mix concentration. The LOD was 2 µM obtained in the absence of any interfering compounds. At 5 µM mix concentration, a detection limit of 3 µM was obtained. When the mix concentration was increased to 25 µM, the detection limit increased to 6 µM and remained the same up to 100 µM mix background.

**Figure 5:**
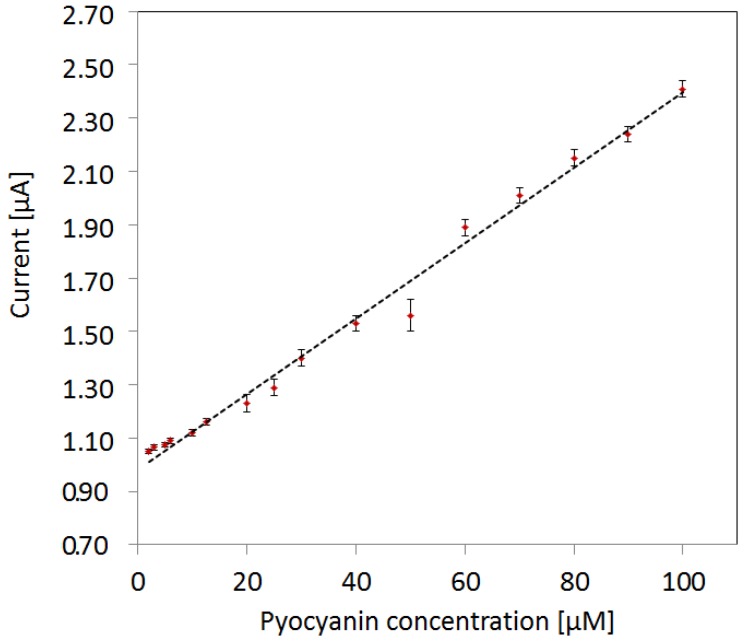
Detection limit of pyocyanin using max peak currents of cyclic voltammograms. The limit of detection (LOD) is 2 µM. The linear fit to peak currents *vs.* pyocyanin concentration has a linearity of 0.991. The error bars represent the standard deviation from measurements in the four different backgrounds of mix concentrations and in no mix background.

The max peak current of pyocyanin as function of concentration has a linearity fit of *R*^2^ = 0.991 (Figure 5). The LOD represents the lowest detectable concentration where a peak was detected by the NOVA1.11 software in the cyclic voltammograms. The LOD is 2 µM, when no mix is present in the samples. The error bars are a measure of the standard deviation of the measurements of pyocyanin in varying mix backgrounds and measurements in MilliQ water. The detection in varying mix backgrounds mimics reality better than using a constant background concentration as the background will always be unknown. The small error bars reveal that pyocyanin can be detected independently by cyclic voltammetry in its respective detection window regardless of the concentration of interfering compounds with an upper detection limit of 6 µM. 

**Figure 6:**
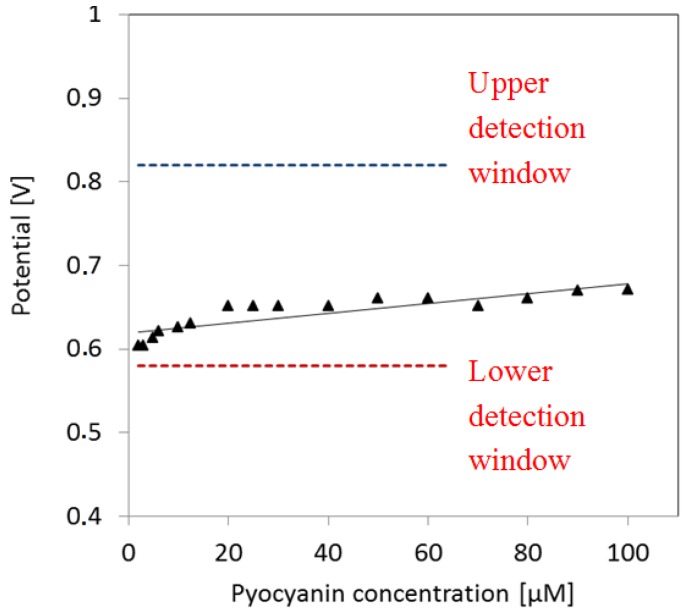
Peak potential as function of pyocyanin concentration. The potential peaks go towards the lower detection window of pyocyanin as the pyocyanin concentration decreases.

The peak potential of pyocyanin shifts slightly to higher values as a function of pyocyanin concentration (Figure 6). The fit indicates a slight increase of potential as a function of concentration. As the pyocyanin concentration decreases, the corresponding potential peak approximates the lower limit of the pyocyanin detection window. This may explain the detection limit around 2 µM.

### 3.4. Detection of Pyocyanin in Saliva Samples

**Figure 7:**
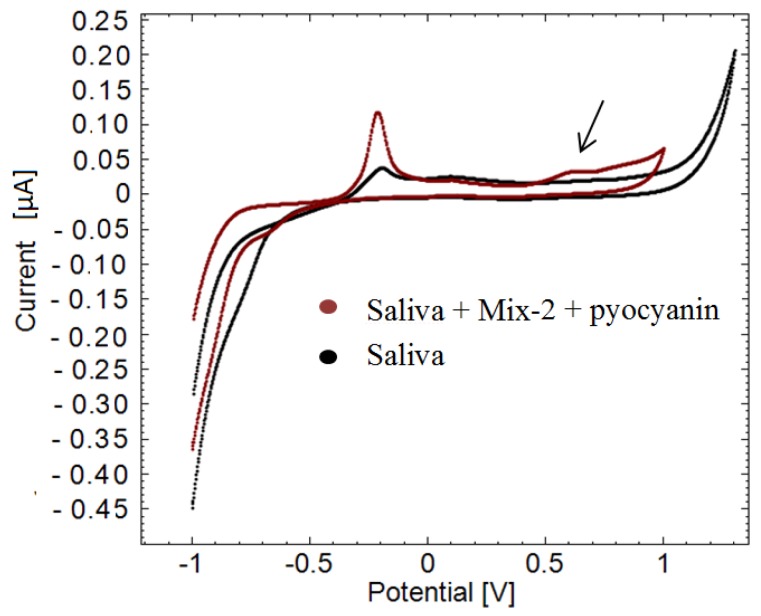
Example of pyocyanin detection in human saliva sample. Black illustrates a cyclic voltammogram of human saliva without pyocyanin. Dark red cyclic voltammogram is of human saliva containing pyocyanin and mix. The peak around 0.60 V *versus* the reference electrode confirms the presence of pyocyanin in the sample (arrow).

An example of a cyclic voltammogram of healthy human saliva is plotted along with a cyclic voltammogram of human saliva containing 160 µM pyocyanin and 50 µM mix-2 in Figure 7. Mix-2 was added in order to complicate the background and observe if it is still possible to detect the pyocyanin. Complicating the background is important since the interference caused by dead cells in the human saliva is not necessarily equal from person to another. Peaks at −0.20 V are seen in both measurements while a peak at 0.60 V only appears in the saliva sample containing pyocyanin. The peak potential lies within the selectivity window of pyocyanin, thus confirming the sensing of pyocyanin. The peak current is 3.31 × 10^−6^, corresponding to 164 ± 2 µM (*n* = 5) pyocyanin according to the calibration curve in Figure 5. It is a deviation of 2.5% ± 1% SE from the known pyocyanin concentration added to the samples.

Earlier studies assume that no interference occurs at negative potentials [13,14,16]. However, the peaks appearing at −0.20 V in the saliva samples can easily interfere with the signals of pyocyanin if a negative detection window is used to detect pyocyanin. Once again, the potential window with a peak at −0.560 V detected in the cyclic voltammogram of pure pyocyanin (Figure 1 and Figure 2) did not appear in the cyclic voltammogram of saliva spiked with pyocyanin in Figure 7. This leaves the optimal reliable detection window for this particular situation to be the positive potential window.

Another peak around 0.11 V is observed in both cyclic voltammograms in Figure 7. This peak might belong to mix compounds as the potential is similar to what was observed in Figure 3. Saliva comprises mainly dead cells that are normally shed from the mucosal surface of the tongue and gums and from the inside of the cheeks [19]. Dead cells release metabolites such as NAD, NADH, NADP and NADPH which might have been detected before being taken up by living bacteria. As the background interference caused by dead cells might differ from patient to patient, the proposed method is an easy way to selectively detect pyocyanin without considering the non-constant background. This is especially beneficial when measuring in sputum samples collected from children, where a significant amount of water and saliva comes up with the expectorate.

The ability to detect pyocyanin at positive potentials is advantageous both with regard to avoiding interfering signals and with regard to enhancing the signal-to-noise ratio [23]. Although the LOD was not as low as in other studies, this work is a proof of concept that it is possible to detect pyocyanin at relatively high positive potentials while preserving the selectivity and speed of sensing. In further studies, the sensors can be optimized and an electrolyte other than MilliQ water can be used to improve the electrochemical detection. As the proof of concept has been established in this work, submicromolar LOD can be investigated using alternative techniques to cyclic voltammetry. For instance, chronoamperomtry is a more sensitive electrochemical quantification tool that is likely to give higher resolution and detection limits due to its high signal-to-noise ratio [24,25].

Saliva is the body fluid closest to sputum. Today sputum samples from cystic fibrosis patients are used for bacterial culturing to identify the type of infection. If the sensors can be used to detect bacteria in sputum samples, it will be possible to avoid lengthy culturing procedures and costly bacterial sequencing. This work demonstrates a direct, sensitive and specific method to determine medically relevant pyocyanin levels in complex samples from patients without any pretreatment procedures [6,26].

## 4. Conclusions

Commercially available, disposable, screen-printed electrodes were used to selectively detect pyocyanin among interfering redox-active compounds. For the first time, a relatively high potential window of 0.58–0.82 V was used to identify pyocyanin in cyclic voltammograms. The advantage of using this potential window is that it lies outside the characteristic potential windows of other interfering compounds that could exist in human samples. The concept was successfully tested on human saliva and artificial sputum, which are the closest fluids to real sputum samples from patients. The detection of biologically important pyocyanin concentrations by disposable electrodes in complex backgrounds opens up the potential application for real-time *in situ* measurements in clinical samples or during surgeries.

## Supplementary Files

Supplementary File 1
