# Supplementary material for: Fast Selective Detection of Pyocyanin Using Cyclic Voltammetry
Source: Sensors (Basel). 2016 Mar 19;16(3):408. doi: 10.3390/s16030408 (PMC4813983; doi:10.3390/s16030408)
Supplement: Supplementary File 1 [file sensors-16-00408-s001.pdf]

Supplementary Materials: Fast Selective Detection of Pyocyanin Using Cyclic Voltammetry

Fatima AlZahra'a Alatraktchi [1,2,3,*], Sandra Breum Andersen [2], Helle Krogh Johansen [2,4], Søren Molin [2,3] and Winnie E. Svendsen [1]

Table S1. Cyclic voltammetry data of pyocyanin.

Index	Peak Position	Peak Height	Peak Area	Base Start	Base End	Peak Width Half	Peak (1/2)	Peak Sum of Derivatives
1	−0.56061	8.3239×10^{-7}	6.5914×10^{-8}	−0.6485	−0.45074	0.074592	0.037663	3.1183×10^{-5}
2	−0.31158	2.6197×10^{-7}	5.6618×10^{-8}	−0.48248	−0.13336	0.22789	0.10694	5.3865×10^{-6}
3	0.69916	4.9116×10^{-7}	6.9902×10^{-8}	0.57953	0.82424	0.13364	0.057625	1.1657×10^{-5}

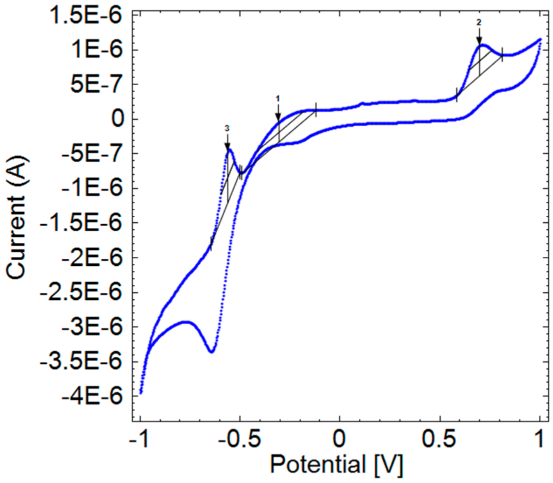

Figure S1. Cyclic voltammetry of pyocyanin.

Table S2. Cyclic voltammetry data of lysogeny broth (LB).

Index	Peak Position	Peak Height	Peak Area	Base Start	Base End	Peak Width Half	Peak (1/2)	Peak Sum of Derivatives
1	0.032654	1.9003×10^{-6}	3.9156×10^{-7}	−0.096741	0.28656	0.20532	0.078073	3.552×10^{-5}
2	−0.81207	1.7701×10^{-6}	2.9382×10^{-7}	−0.8902	−0.62408	0.17323	0.059056	7.4477×10^{-5}

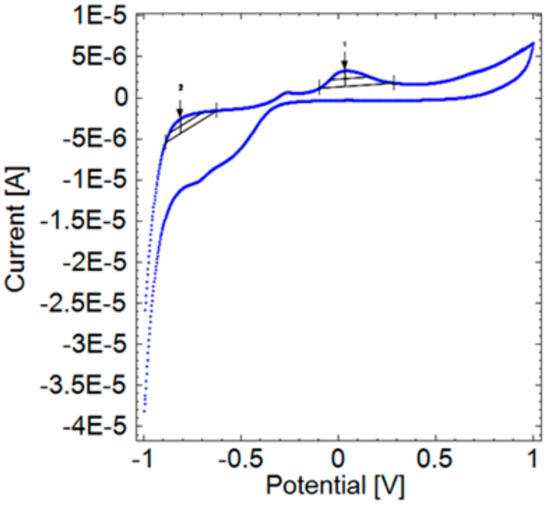

Figure S2. Cyclic voltammogram of lysogeny broth (LB).

Table S3. Cyclic voltammetry data of pyoverdine.

Index	Peak Position	Peak Height	Peak Area	Base Start	Base End	Peak Width Half	Peak (1/2)	Peak Sum of Derivatives
1	0.093689	2.9181×10^{-7}	1.259×10^{-7}	−0.22858	0.52582	0.4377	0.19638	3.0855×10^{-6}

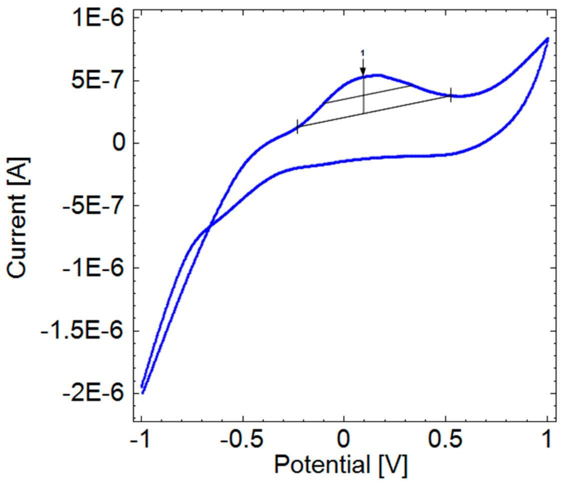

Figure S3. Cyclic voltammogram of pyoverdine.

Table S4. Cyclic voltammetry data of NADP.

Index	Peak Position	Peak Height	Peak Area	Base Start	Base End	Peak Width Half	Peak (1/2)	Peak Sum of Derivatives
1	0.049744	1.488×10^{-7}	4.1638×10^{-8}	−0.19928	0.26215	0.28683	0.15006	2.1831×10^{-6}

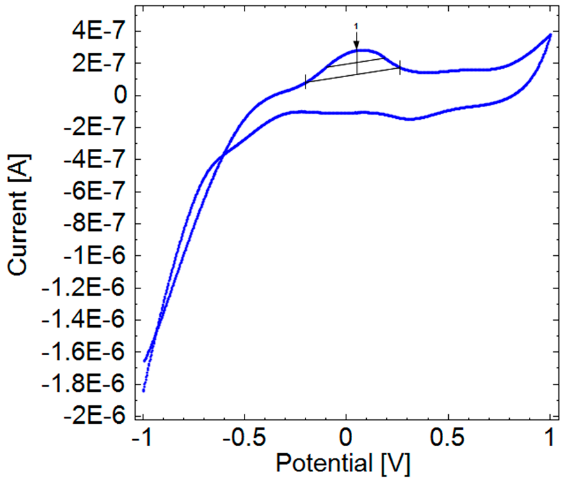

Figure S4. Cyclic voltammogram of NADP.

Table S5. Cyclic voltammetry data of NADH.

Index	Peak Position	Peak Height	Peak Area	Base Start	Base End	Peak Width Half	Peak (1/2)	Peak Sum of Derivatives
1	0.015564	4.1348×10^{-7}	1.4664×10^{-7}	−0.29205	0.23671	0.34932	0.1771	5.6019×10^{-6}
2	0.410692	1.4328×10^{-7}	0.8431×10^{-8}	0.24965	0.58929	0.05782	0.0671	2.1417×10^{-6}

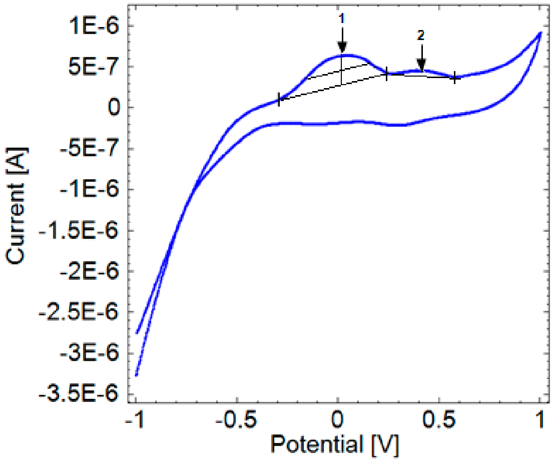

Figure S5. Cyclic voltammogram of NADH.

Table S6. Cyclic voltammetry data of NADPH.

Index	Peak Position	Peak Height	Peak Area	Base Start	Base End	Peak Width Half	Peak (1/2)	Peak Sum of Derivatives
1	0.0082398	4.4315×10^{-7}	1.6184×10^{-7}	−0.27496	0.2787	0.36127	0.15333	5.0112×10^{-6}
2	0.487830	0.9348×10^{-7}	0.4664×10^{-8}	0.334434	0.58038	0.06932	0.07871	1.6049×10^{-6}

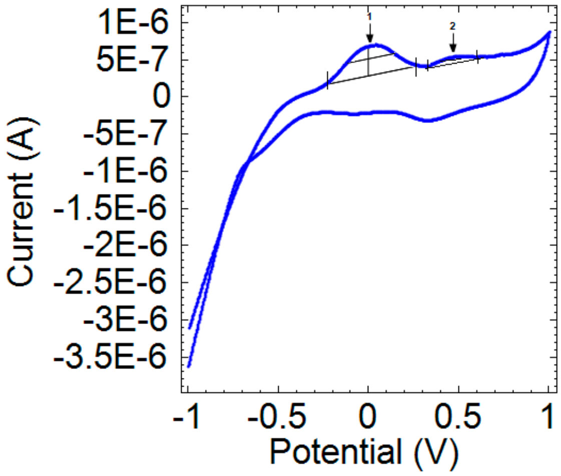

Figure S6. Cyclic voltammogram of NADPH.

Table S7. Cyclic voltammetry data of phenazine-$C_{12}H_8N_2$.

Index	Peak Position	Peak Height	Peak Area	Base Start	Base End	Peak Width Half	Peak (1/2)	Peak Sum of Derivatives
1	0.17426	1.471×10^{-7}	5.6218×10^{-8}	−0.16266	0.51361	0.37566	0.18177	1.3341×10^{-5}

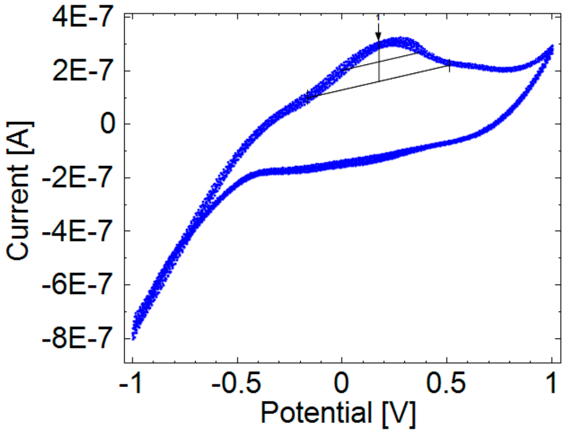

Figure S7. Cyclic voltammogram of phenazine-$C_{12}H_8N_2$.

Table S8. Cyclic voltammetry data of artificial sputum (ASM).

Index	Peak Position	Peak Height	Peak Area	Base Start	Base End	Peak Width Half	Peak (1/2)	Peak Sum of Derivatives
1	−0.22858	7.0743×10^{-6}	5.1823×10^{-7}	−0.38238	−0.14069	0.063384	0.036463	0.0003219

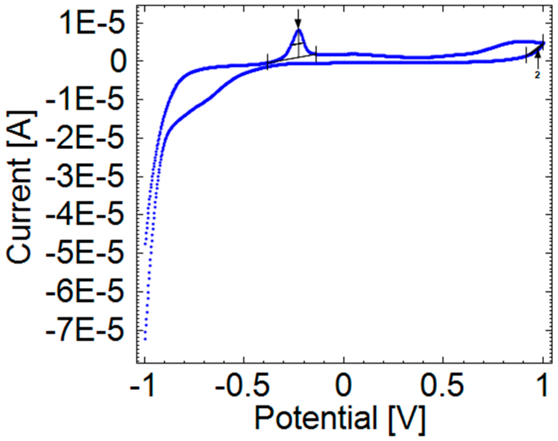

Figure S8. Cyclic voltammogram of artificial sputum (ASM).

Table S9. Cyclic voltammetry data of human saliva.

Index	Peak Position	Peak Height	Peak Area	Base Start	Base End	Peak Width Half	Peak (1/2)	Peak Sum of Derivatives
1	−0.20904	1.084×10^{-5}	1.098×10^{-6}	−0.40192	0.00091553	0.080447	0.039377	0.00037927

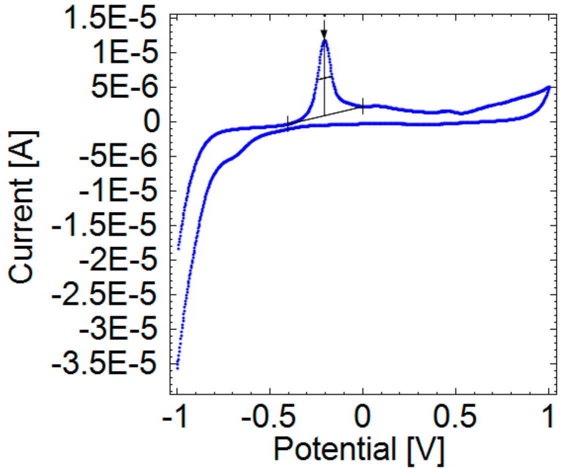

Figure S9. Cyclic voltammogram of human saliva.
